# Methodological issues on “Stakeholder attitudes to the regulation of traditional and complementary medicine professions: a systematic review”

**DOI:** 10.1186/s12960-022-00718-z

**Published:** 2022-03-09

**Authors:** Meysam Shirzad, Alireza Abbassian

**Affiliations:** Department of Traditional Medicine, School of Persian Medicine, Tehran University of Medical Sciences, Tehran, Iran

**Keywords:** Traditional and complementary medicine, Stakeholders, Systematic review, Persian medicine, Uyghur medicine

## Abstract

Systematic reviews cling to the doctrine that science has an updating databank and attempt to identify all available evidence by featured eligibility criteria to find the answer to a unique scientific question. Therefore, to reach this aim, these researches should use a wise method and comprehensive search strategy, as they are widely used to guide clinical and political decisions and the establishment of future researches. We would like to appreciate Jenny Carè, Amie Steel, and Jon Wardle for the valuable article “Stakeholder attitudes to the regulation of traditional and complementary medicine professions: a systematic review”. Some important missed search terms in the field of traditional medicine names and traditional and complementary medicine (T&CM) regulation concepts were discussed in the article.

To the Editor

One of the most consequential parts of systematic reviews is search terms determination and related search strategy. Systematic reviews stick to the rule that science has an updating dataset and try to identify all related evidence by featured eligibility criteria to find the answer to a unique scientific question. Therefore, to reach this goal, these researches should follow a wise method leading to a comprehensive search strategy, as they are widely used to guide clinical and political decisions and the establishment of future researches. We read with interest the systematic review article by Carè et al. on the stakeholder attitudes to the regulation of traditional and complementary medicine (T&CM) professions [1]. In the search protocol which is fully available at: https://www.crd.york.ac.uk/PROSPEROFILES/198767_STRATEGY_20200714.pdf, T&CM terms are listed in concept 1, and regulation terms are listed in concept 2 table. These two lists provided informative terms for the search strategy. However, it seems that both of them missed some important terms and expressions. For instance, in the concept 1 list, Persian medicine, and Uyghur medicine as two important traditional medicines might have been put in the list:

In a gross search in the PubMed database with the term ‘Persian medicine’ (in tile/abstract), 389 records returns and 297 records returns for ‘Iranian traditional medicine’ (another synonym of Persian medicine).

About Uyghur medicine, the numbers are 62 records for ‘Uyghur medicine’, 72 records for ‘Uighur medicine’, 3 for ‘Uigur medicine, and 32 for ‘Uygur medicine’ dictation.

As a comparable example, Unani medicine, which is present currently in concept 1 table, has 165 records in the same database, but Persian medicine has 686 records (sum of 389 and 297) and Uyghur medicine has 169 records (sum of deferent dictations). Therefore, as a search term, Persian medicine and Uyghur medicine should be included in the concept 1 list.

Also, both of these terms have published investigations on regulation [2–4]. Moreover, according to the World Health Organization (WHO) Traditional Medicine Strategy 2014–2023, some terms such as registration, integration, and classification are main milestones for the regulation of T&CM professions and could be inserted in the concept 2 list [5].

## Conclusions

The related discussed article needs some updates in search terms which are listed in the different concepts.

## Data Availability

Not applicable.

## Abbreviations

T&CM: Traditional and complementary medicine
WHO: World health organization
